# miR-let-7 Targeting *ZcCTL-S1* to Regulate Reproductive Development in *Zeugodacus cucurbitae*

**DOI:** 10.3390/insects17030286

**Published:** 2026-03-05

**Authors:** Yi-Kun Zhang, Guo-Feng Zhang, Li-Xiang Chen, Yu-Xue Zhang, Shi-Yuan Wang, Ke-Qing Deng, Lai-Wai Tun, Zhong-Shi Zhou, Lu Peng

**Affiliations:** 1 State Key Laboratory of Agricultural and Forestry Biosecurity, Institute of Applied Ecology, Fujian Agriculture and Forestry University, Fuzhou 350002, China; 15839424238@163.com (Y.-K.Z.); windlycountry@outlook.com (G.-F.Z.); clx024028@163.com (L.-X.C.); zhangyuxue1219@163.com (Y.-X.Z.); wangshiyuan719@163.com (S.-Y.W.); dengkeqing2906@163.com (K.-Q.D.); laewaitun.lwt12@gmail.com (L.-W.T.); 2 State Key Laboratory for Biology of Plant Diseases and Insect Pests, Institute of Plant Protection, Chinese Academy of Agricultural Sciences, Beijing 100193, China; 3 National Nanfan Research Institute, Chinese Academy of Agricultural Sciences, Sanya 572019, China; 4 Ministerial and Provincial Joint Innovation Centre for Safety Production of Cross Strait Crops, Fujian Agriculture and Forestry University, Fuzhou 350002, China; 5 Key Laboratory of Integrated Pest Management for Fujian-Taiwan Crops, Ministry of Agriculture, Fuzhou 350002, China; 6 Fujian Provincial Key Laboratory of Insect Ecology, Fujian Agriculture and Forestry University, Fuzhou 350002, China

**Keywords:** *Zeugodacus cucurbitae*, *ZcCTL-S1*, miR-let-7, hatching rate, reproductive regulation

## Abstract

The melon fly, *Zeugodacus cucurbita*e (Coquillett), is recognized as a globally significant quarantine pest, and it ranks among the most destructive insect species infesting cucurbit and solanaceous crops. However, the molecular mechanisms governing reproductive regulation in female *Z. cucurbitae* remain poorly characterized, particularly those underlying the reproductive processes mediated by microRNAs (miRNAs). In this study, we firstly identified the ovary-specific gene *ZcCTL-S1* in *Z. cucurbitae* via transcriptomic analysis, and subsequently predicted its targeted miRNAs using bioinformatics approaches. Among these miRNAs, overexpression or inhibition of miR-971-1 and miR-let-7 led to corresponding inverse changes in the transcriptional level of *ZcCTL-S1*. Notably, only miR-let-7 displayed markedly elevated expression levels in *Z. cucurbitae* ovaries. Further analyses confirmed that miR-let-7 exhibited a direct targeting relationship with *ZcCTL-S1*, via a combinatorial approach involving in vivo RNA immunoprecipitation, in vitro dual-luciferase reporter assays, and site-directed mutagenesis techniques. Phenotypic analyses showed that both knockdown of *ZcCTL-S1* and overexpression of miR-let-7 significantly inhibited egg hatchability, ultimately compromising the female reproductive capacity of *Z. cucurbitae*. Collectively, these findings identify a novel miRNA-gene regulatory module in the reproductive development of *Z. cucurbitae*, and provide novel insights for the development of gene- or miRNA-based pest control strategies.

## Simple Summary

The molecular mechanisms governing reproductive regulation in female *Zeugodacus cucurbita*e remain poorly characterized, particularly those underlying the reproductive processes mediated by microRNAs (miRNAs). Our study identifies a novel miRNA-gene regulatory module consisting of miR-let-7 and the ovary-specific C-type lectin gene *ZcCTL-S1*, which is indispensable for female fecundity in *Z. cucurbitae*. These findings provide a deeper insight for the development of gene- or miRNA-based pest control strategies.

## 1. Introduction

The melon fly, *Zeugodacus cucurbitae* (Coquillett), is recognized as a globally significant quarantine pest, and it ranks among the most destructive insect species infesting cucurbit and solanaceous crops across the Pacific, Asia, and Africa [1,2]. Female adults use their ovipositors to lay eggs beneath the pericarp, and the resulting larvae feed on internal fruit tissues, causing rot and reducing crop yield and quality [3]. The larvae’s specialized feeding habits, combined with pesticide resistance of adults, reduce the effectiveness of conventional chemical control strategies and increase the likelihood of secondary problems, including environmental pollution and pesticide residues in agricultural produce. Thus, the formulation of targeted and environmentally benign management strategies, including the suppression of reproductive potential via genetic approaches, is essential for controlling the population growth of *Z. cucurbitae*.

C-type lectins (CTLs) are widely distributed carbohydrate-binding proteins within the animal lectin family and display Ca^2+^-dependent carbohydrate recognition activity [4]. Most CTLs consist of a signal peptide, a Ca^2+^-binding domain, and a C-type lectin domain (CTLD); among these, the CTLD serves as the functional region that mainly supports sugar recognition and binding [5]. In insects, C-type lectins are structurally divided into three major classes: CTL-S, characterized by a single CRD; immulectins, which contain tandem CRDs; and CTL-X, which exhibit additional or complex domain architectures. Notably, CTL-X lectins harbor multiple functional domains, including complement control protein (CCP), immunoglobulin (Ig) modules, and epidermal growth factor-like (EGFL) domains [5,6]. CTL proteins are broadly expressed across diverse insect species and are commonly regarded as pivotal to pathogen recognition and immune defense [7]. This is attributable to the CTL’s core CRD structure, which recognizes pathogen-associated molecular patterns (PAMPs), including lipopolysaccharides (LPS), peptidoglycan (PGN), and β-glucan, thereby triggering immune responses [7,8]. For instance, in *Ostrinia furnacalis* (Lepidoptera: Pyralidae) and *Bombyx mori* (Lepidoptera: Bombycidae), CTLs can recognize LPS and upregulate antimicrobial peptide expression; in certain contexts, they also drive melanization by activating the phenol oxidase (PO) cascade, thereby enhancing survival rates [6,8,9,10]. Likewise, in *Helicoverpa armigera* (Diptera: Noctuidae), C-type lectins interact with β-integrin to promote hemocyte encapsulation and bolster immune defenses [11].

However, recent studies indicate that CTL-mediated Ca^2+^-dependent carbohydrate recognition plays a role in various aspects of insect reproduction, such as ovarian maturation, gamete binding, and embryonic development [12]. Insects also depend on CTLs for immune defense within the reproductive tract and in body fluids. For instance, in *Tribolium castaneum* (Coleoptera: Tenebrionidae), *TcCTL9* knockdown reduces egg hatchability by impairing embryogenesis and immune function [13]. Similarly, female *H. armigera* enhance their antibacterial defenses through CTLs, thereby supporting successful fertilization [14]. These findings indicate that CTLs not only maintain immune homeostasis in insects but are also deeply involved in reproductive regulation through multiple mechanisms, providing new insights for pest control and biotechnological applications [8,15,16,17]. However, it remains unknown whether ovary-specific members of the CTL family regulate reproductive development in *Z. cucurbitae* through upstream regulatory factors, such as microRNAs (miRNAs).

miRNAs are endogenous non-coding RNAs of roughly 22 nucleotides that function as post-transcriptional regulators of gene expression. They were first reported in *Caenorhabditis elegans* (Rhabditida: Caenorhabditidae) in 1993 [18]. miRNAs do not encode proteins but can regulate gene expression by targeting messenger RNA (mRNA). Their primary mechanisms include mRNA degradation and translation repression, through which they modulate gene expression [19,20]. Accumulating studies indicate that miRNAs influence core reproductive processes in insects (e.g., ovarian development, egg maturation, and germ cell maintenance) via the targeted regulation of specific genes [21]. Evidence from insect studies highlights the crucial role of miRNAs in reproductive regulation. In *Bactrocera dorsalis* (Diptera: Tephritidae), miR-31b has been experimentally confirmed to target *arylsulfatase B*, thereby altering metabolic homeostasis and suppressing ovarian development [22]. Likewise, the knockdown of essential miRNA pathway genes, Ago1 and Dcr1, reduces miR-let-7 and miR-184 abundance, leading to impaired fecundity, fertility, and survival in *Bemisia tabaci* (Diptera: Aleyrodidae) [23]. Collectively, these findings emphasize the scientific value of investigating miRNA–target interactions in reproductive development.

Herein, we initially identified the ovary-specific C-type lectin gene *ZcCTL-S1* using transcriptomics, and subsequently predicted its target miRNAs through bioinformatics analyses. The binding interaction between *ZcCTL-S1* and candidate miRNAs was validated in vivo via RNA immunoprecipitation assays and in vitro using a dual-luciferase reporter system. Furthermore, site-directed mutagenesis was used to precisely pinpoint the core binding sites of miRNA within *ZcCTL-S1*. Both *ZcCTL-S1* knockdown and overexpression of miR-let-7 resulted in a significant reduction in the fecundity of *Z. cucurbitae*. Overall, this study advances our understanding of the transcriptional regulatory mechanisms governing female reproductive functions in this pest species. Furthermore, it provides novel insights for the development of gene- or miRNA-based pest control strategies, thereby highlighting the substantial theoretical significance and practical utility of our findings.

## 2. Materials and Methods

### 2.1. Insects

A laboratory strain of *Z. cucurbitae* was reared in a controlled artificial climate chamber (26 ± 1 °C, 50% relative humidity, 14:10 h light/dark cycle) at the State Key Laboratory of Agricultural and Forestry Biosecurity, Fujian Agriculture and Forestry University. Larvae were fed with fresh *Cucurbita pepo* L. until pupation, with pupae subsequently transferred to moist sand to facilitate adult emergence. Newly emerged adults were supplied an artificial diet of yeast extract and sucrose 2:1, *w*/*w*, and eggs laid on *C. pepo* slices were collected daily to propagate subsequent generations.

### 2.2. Transcriptome Sequencing

Different tissues, including ovaries, fat bodies, midguts, and Malpighian tubules, were dissected from 10-day-old virgin females under a stereomicroscope (Leica, Wetzlar, Germany), with 20–30 individuals and three replicates in each treatment. Total RNA was extracted using the Eastep^®^ Super Total RNA Extraction Kit (Promega, Madison, WI, USA), and its integrity and concentration were assessed with an Agilent 2100 Bioanalyzer (Agilent Technologies, Santa Clara, CA, USA). A measure of 10 μg of RNA was used for sequencing library construction with the NEBNext^®^ Ultra™ RNA Library Prep Kit (New England Biolabs, Ipswich, UK). Subsequently, paired-end sequencing was performed on the Illumina NovaSeq 6000 by Novogene Bioinformatics Technology (Beijing, China). Raw sequencing data were filtered using Fastp v0.23.1 to obtain high-quality clean reads, with key quality metrics (Q20, Q30, and GC content) calculated simultaneously. The clean reads were aligned to the *Z. cucurbitae* reference genome using Hisat2 v2.0.5 under default parameters, followed by transcript assembly via StringTie v1.3.3b. For gene expression quantification, FeatureCounts v1.5.0-p3 was employed. Differentially expressed genes (DEGs) were identified using DESeq2 v1.20.0 with the thresholds of |log_2_fold change (FC)| ≥ 1 and *p* ≤ 0.05. Subsequently, functional annotation of DEGs was performed against the Gene Ontology (GO) and Kyoto Encyclopedia of Genes and Genomes (KEGG) databases.

### 2.3. RNA Extraction, cDNA Synthesis, and Gene Cloning

Total RNA was isolated from individual samples or corresponding tissues with the SteadyPure RNA Extraction Kit (Promega, Madison, WI, USA) in accordance with the manufacturer’s standard protocols. The integrity and concentration of the extracted RNA were subsequently quantified and verified using a NanoVue spectrophotometer (GE Healthcare, Chicago, IL, USA). For cDNA synthesis, 1 μg of the qualified RNA was reverse-transcribed by Evo M-MLV Reverse Transcriptase (Accurate Biology, Changsha, China). The target gene *ZcCTL-S1* was amplified via PCR with gene-specific primers (Table S1) and Phanta Max Super-Fidelity DNA Polymerase (Vazyme, Nanjing, China), following the thermal cycling procedure of treatment at 95 °C for 3 min, followed by 34 cycles of 95 °C for 30 s, 58 °C for 30 s, and 72 °C for 60 s, followed by a final 5 min step at 72 °C.

### 2.4. Molecular Characterization and Phylogenetic Analysis of ZcCTL-S1

The candidate nucleotide sequence of *ZcCTL-S1* was retrieved from the NCBI GenBank database (https://www.ncbi.nlm.nih.gov/, accessed on 23 June 2025) under accession number LOC105210885. Conserved domains were analyzed using the CD-Search tool (https://www.ncbi.nlm.nih.gov/Structure/cdd/wrpsb.cgi, accessed on 23 June 2025) and the presence of a signal peptide was predicted withSignalP-6.0 (https://services.Healthtech.dtu.dk/services/SignalP-6.0/, accessed on 23 June 2025). Additionally, potential N-glycosylation sites and putative phosphorylation sites were predicted using NetNGlyc 1.0 and NETPHOS 3.1, respectively, through the CBS prediction server (http://www.cbs.dtu.dk/services, accessed on 23 June 2025). The corresponding amino acid sequence was translated and confirmed using the translation function in Snap Gene 4.1.9 (Dotmatics, Boston, MA, USA). To identify homologs, the validated ZcCTL-S1 protein sequence was subjected to BLASTP against protein sequences from eight related insect species, including *Bactrocera oleae* (Diptera: Tephritidae), *B. dorsalis*, *Bactrocera tryoni* (Diptera: Tephritidae), *Aedes aegypti* (Diptera: Culicidae), *Anopheles albimanus* (Diptera: Culicidae), *Anopheles coluzzii* (Diptera: Culicidae), *Calliphora vicina* (Diptera: Calliphoridae), and *Lucilia sericata* (Diptera: Calliphoridae). A neighbor-joining (NJ) phylogenetic tree was constructed using MEGA-64 software with 1000 bootstrap replications for reliability assessment. Subsequently, the generated phylogenetic tree was visually optimized using ChiPlot (https://www.chiplot.online/#, accessed on 23 June 2025) to enhance readability [24].

### 2.5. miRNAs Prediction

To identify potential miRNA binding sites within the 3′-untranslated region (3′-UTR) and coding sequence (CDS) of the *ZcCTL-S1* gene, a combinatorial bioinformatics approach was employed by integrating predictions from two complementary algorithms, miRanda and RNAhybrid. The insect miRNA reference dataset utilized in this analysis was retrieved from the InsectBase 2.0 database (http://v2.insect-genome.com/miRNA, accessed on 25 June 2025). To ensure the reliability and specificity of predicted interactions, only binding sites consistently identified by both miRanda and RNAhybrid were retained as candidate miRNA-*ZcCTL-S1* pairs.

### 2.6. Expression Profiling of ZcCTL-S1 and Predicted miRNAs

To determine the stage-specific expression profiles of the target genes and candidate miRNAs, samples were collected from distinct developmental stages, including eggs, larvae at 1, 3, and 7 days, pupae at 1, 5, and 9 days, and female adults at 1, 3, 5, 7, and 9 days post-emergence. For each stage, three biological replicates were prepared, and each replicate contained approximately 20 mg of sample tissue.

For tissue-specific expression analysis, 5 d old female adults were dissected to isolate four tissues (ovary, fat body, midgut, and Malpighian tubules). Each dissected tissue was immediately immersed in RNA Later (Accurate Biology, Changsha, China) and stored at −80 °C to preserve RNA integrity. For mRNA quantification, total RNA extraction and cDNA synthesis were performed as described in Section 2.3. RT-qPCR analysis was then conducted using the GoTag^®^ qPCR Master Mix Kit (Promega, USA) under the following conditions: initial denaturation at 95 °C for 600 s, followed by 40 cycles of 95 °C for 15 s and 60 °C for 60 s. For miRNA expression analysis, total miRNA was isolated using the miRNA Isolation Kit (Omega, Norcross, GA, USA) and converted to cDNA with the miRNA First-Strand cDNA Synthesis SuperMix (TransGen Biotech, Beijing, China).

The RT-qPCR analysis was conducted using the same cycling parameters described above. Ribosomal protein L13 (RPL13) and U6 were used as internal reference genes for mRNA and miRNA normalization, respectively, and the corresponding primers are provided in Table S1.

### 2.7. Dual-Luciferase Assay

To validate the predicted miRNA-*ZcCTL-S1* binding interactions, specific primers (including primers for the binding site-mutated construct) were designed based on the predicted miRNA-mRNA binding site of the *ZcCTL-S1* gene and its flanking sequences. Primers for a binding site–mutated fragment were also included. Using these primers, dual-luciferase reporter plasmids targeting *ZcCTL-S1* were generated according to the protocol of the pmirGLO Dual-Luciferase miRNA Target Expression Vector (Promega, USA). Briefly, wild-type (WT) and mutant (MUT) fragments containing the predicted miRNA binding site were amplified and inserted into the pmirGLO vector (Promega, USA) via the SacI and SalI restriction sites. This resulted in three constructs: WT-pmirGLO, MUT-pmirGLO, and the empty pmirGLO vector, which served as a negative control. HEK293T cells were then co-transfected with 100 μM of either the miRNA agomir or the agomir negative control (NC), together with 500 ng of WT-pmirGLO, MUT-pmirGLO, or empty pmirGLO plasmid. At 24 h post-transfection, Luciferase Assay Reagent II was added to the cells, and the activities of firefly luciferase (reporter) and Renilla luciferase (internal control) were measured sequentially using a luciferase assay system.

### 2.8. RNA Immunoprecipitation Assays

Three-day-old female adults were microinjected with 250 nL of 100 μM miR-let-7 mimic or mimic negative control (mimic-NC). At 48 h post-injection, ovaries were dissected and analyzed using an RNA-Binding Protein Immunoprecipitation Kit (Absin, China) according to the manufacturer’s instructions. Anti-Ago1 antibody (30 μg) was incubated with magnetic beads, and normal rabbit IgG (30 μg) served as a negative control. Co-immunoprecipitation RNA was purified and analyzed by reverse transcription-quantitative PCR (RT-qPCR) to determine the enrichment level of *ZcCTL-S1* mRNA.

### 2.9. Fluorescence In Situ Hybridization

To visualize the cellular spatial localization of *ZcCTL-S1* and miR-let-7, fluorescence in situ hybridization (FISH) was conducted. The FAM (Carboxyfluorescein)-labeled *ZcCTL-S1* probe and Cy3 (sulfoCyanine3)-labeled miR-let-7 probe were synthesized by Zoonbio Biotechnology (Nanjing, China) (Supporting Information, Table S1). Ovaries were dissected from 5-day-old adult female *Z. cucurbitae* in phosphate-buffered saline (PBS), embedded in paraffin for sectioning, hybridized with probe-loaded hybridization buffer, counterstained with DAPI, and observed under a fluorescence microscope (Nikon, Tokyo, Japan).

### 2.10. RNA Interference and miRNA Overexpression

Gene-specific primers harboring the T7 promoter were designed via NCBI Primer-Blast. Double-stranded RNA (dsRNA) was synthesized using the T7 RiboMAX™ Express RNAi System (Promega, USA), with template amplification conducted under the following conditions: initial denaturation at 95 °C for 3 min, followed by 35 cycles of 95 °C for 30 s, 58 °C for 30 s, and 72 °C for 1 min, and a final extension at 72 °C for 5 min (Table S1). Subsequently, PCR products were purified using the HiPure Gel Pure DNA Mini Kit (Magen Biotechnology, Guangzhou, China). dsRNA was transcribed from *ZcCTL-S1* and *EGFP* templates using the T7 RiboMAX™ Express RNAi System (Promega, USA). The synthesized dsRNA was adjusted to a concentration of 2000 ng/μL, and 1000 nL of ds*CTL-S1* or ds*EGFP* was injected into 3-day-old female adults using a Nanoliter 2010 syringe (WPI, Sarasota, FL, USA). Injections were carried out every 48 h, and the treatment was repeated continuously until the insects reached the 11-day-old larval stage.

Injected female adults were sampled at 24, 48, and 72 h post-injection. Each time point included three biological replicates with four individuals per replicate. For miRNA overexpression, 250 nl of either a 100 μM miRNA mimic or a negative control (NC) was injected following the same protocol as described in Section 2.8.

### 2.11. Phenotypic Observations and Bioassays

Fifteen dsRNA-treated females were mass mated with an equal number of untreated males. Over a period of three consecutive days, the total number of eggs laid and the egg hatching rate were carefully recorded for each mating pair, with three biological replicates. Additionally, their ovaries were then dissected in 1× PBS buffer under a stereomicroscope (Leica, Wetzlar, Germany). Ovarian sizes were measured, and morphological images were acquired using a digital microscope (VHX-2000C, Keyence, Osaka, Japan). A total of 10 replicates per treatment group were analyzed. For the daily laid eggs of *Z. cucurbitae*, egg length was measured individually, and corresponding images were captured using the aforementioned digital microscope, with 20 replicates per group.

### 2.12. Statistical Analysis

Statistical analyses were conducted with IBM SPSS Statistics 21.0 software (SPSS Inc., USA). All experimental data were shown as the mean ± standard error (SE). Student’s *t*-test was applied to evaluate the statistical differences between the ds*CTL-S1* and ds*EGFP* treatment groups, as well as between the miRNA mimic/inhibitor groups and their corresponding negative controls (NC). For comparisons of mRNA and miRNA expression levels across distinct developmental stages and tissues, together with the outcomes of the dual-luciferase reporter assay, one-way analysis of variance (ANOVA) was performed, followed by Tukey’s post hoc test for multiple comparisons. All graphical visualizations of the data were generated using GraphPad Prism 10.5.0.

## 3. Results

### 3.1. Transcriptome Analysis and Candidate Gene Identification

**Figure 1:**
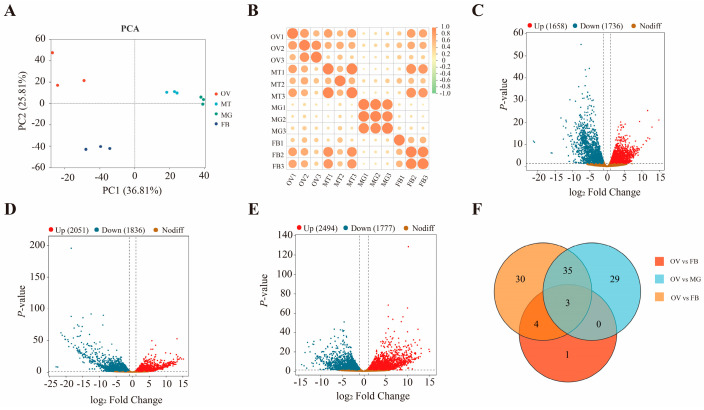
Transcriptome analysis of differentially expressed genes (DEGs) in the ovary, Malpighian tubule, midgut, and fat body of adult female *Z. cucurbitae*. (**A**) Scatter plots depicting PC1 versus PC2 derived from the principal component analysis. (**B**) Correlation analysis among different samples. The size of the circles represents the magnitude of the R^2^ values; the larger the circle, the larger the value. (**C**–**E**) DEGs between the OV and FB, MG, and MT. (**F**) Venn diagram of sample gene co-expression, showing the number of extremely upregulated genes in the ovary (|log2fold change (FC)| > 10). Here, OV, FB, MT, and MG represent the ovary, fat body, Malpighian tubules, and midgut, respectively.

Four samples generated ≥ 45.37 Gb clean reads, with Q30 values ≥ 96.61% and GC contents ranging from 40.45 to 44.5% (Table S2). Clean reads from each sample were mapped to the reference genome, with mapping rates of approximately 90% across all tissues except the midgut, which had a mapping rate of < 60% (Table S2). In a principal component analysis, the samples for each tissue clustered well along the first principal component (PC1) and second principal component (PC2), confirming that these biological replicates exhibited good repeatability and that there were clear differences in the gene expression profiles of different tissues (Figure 1A). Pearson’s correlation coefficient squared (R^2^) between all biological replicates exceeded 0.90, indicating high reproducibility (Figure 1B). A total of 3394 differentially expressed genes (DEGs) were detected in the ovary relative to the fat body, among which 1658 were upregulated, and 1736 were downregulated (Figure 1C). Similarly, 3887 DEGs were identified in the ovary versus the midgut, with 2051 genes showing upregulation and 1836 showing downregulation (Figure 1D). Furthermore, comparison of the ovary with the Malpighian tubule revealed 4271 DEGs, including 2494 upregulated and 1777 downregulated genes (Figure 1E). Among these, three genes exhibiting extremely high upregulation in the ovary relative to other tissues were identified with |log2fold change (FC)| > 10 (Figure 1F). Ten genes were randomly selected for RT-qPCR verification, and their expression patterns were consistent with the transcriptome analysis. (Figure S1).

### 3.2. ZcCTL-S1 Genes Characteristics and Phylogenetic Analysis

**Figure 2:**
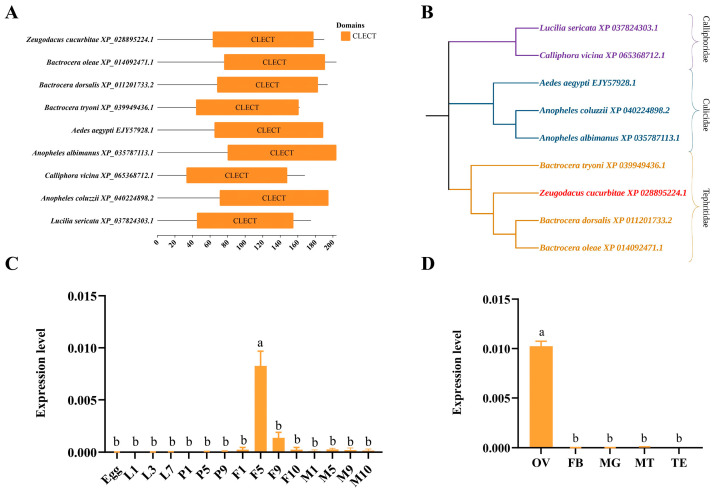
Conserved domains, phylogenetic tree of insect ZcCTL-S1 proteins, and expression patterns of *ZcCTL-S1* gene across different developmental stages and tissues. (**A**) Conserved domains of insect CTLs. The conserved domains of CTLs were visualized using Tbtools. (**B**) Phylogenetic tree of CTL proteins. The phylogenetic tree was constructed by the neighbor-joining method using MEGA 64 software. (**C**) Relative expression level of *ZcCTL-S1* at different developmental stages. (**D**) Relative expression level of *ZcCTL-S1* in different tissues. Egg: egg; L1/3/7: 1st/3rd/7th instar larva; P1/5/9: 1st/5th/9th instar pupa; 1/3/5/7/9/10F: 1st/3rd/5th/7th/9th/10th instar female adult; 1/5/9/10M: 1st/5th/9th/10th instar male adult; OV: ovary; FB: fat body; MG: midgut; MT: Malpighian tubule. Data were analyzed using a one-way analysis of variance followed by Tukey’s test. Different letters indicate significant differences (*p* < 0.05).

The full-length coding sequence (CDS) of *ZcCTL-S1* is 573 bp, encoding a protein of 190 amino acids (Figure S2). A signal peptide was predicted at the N-terminus from amino acids 1–21, with a cleavage site identified between amino acids 21 and 22 (after Ala) (Figure S3A). The ZcCTL-S1 protein was predicted to harbor two glycosylation sites (Figure S3B), along with 33 phosphorylation sites (Figure S3C). Conserved domain analysis demonstrated that the C-type lectins (CTLs) from nine insect species share an identical conserved domain, namely the CLECT domain (Figure 2A). Phylogenetic analysis categorized these CTLs into three distinct clades, with the clustering pattern largely matching the taxonomic families of the corresponding insect species. Notably, *Z. cucurbitae* showed the closest genetic affinity to the members of the family Tephritidae (Figure 2B).

### 3.3. ZcCTL-S1 Gene Expression Patterns

*ZcCTL-S1* expression was initiated as early as in newly emerged female adults, displaying a trend of initial upregulation followed by subsequent downregulation, with peak expression levels detected in 5-day-old female adults (*F*_(14,22)_ = 32.4029, *p* = 0.0000) (Figure 2C). Moreover, the tissue-specific expression analysis revealed that *ZcCTL-S1* was predominantly expressed in the ovaries, and its expression level here was significantly higher than that in the fat body, midgut, and Malpighian tubules (*F*_(3,7)_ = 360.5106, *p* = 0.0000) (Figure 2D). FISH assays revealed that the hybridization signal of the *ZcCTL-S1*-specific probe was predominantly localized in the trophocytes and oocytes.

### 3.4. Effects of ZcCTL-S1 Knockdown on Reproduction

**Figure 3:**
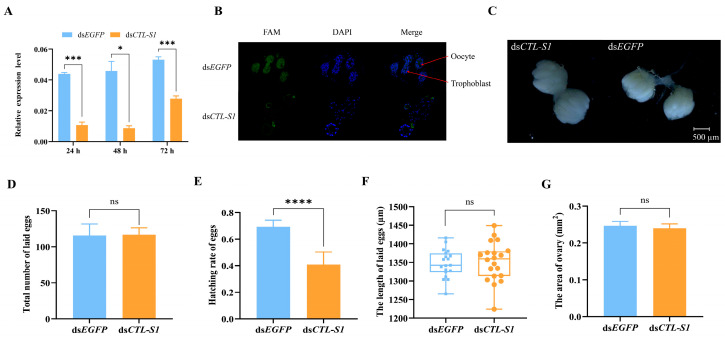
Effects of *ZcCTL-S1* knockdown on the development and reproduction of *Z. cucurbitae.* (**A**) Relative expression level of *ZcCTL-S1* after ds*CTL-S1* injection. (**B**) Fluorescence in situ hybridization (FISH) localization of *ZcCTL-S1* in the ovary of *Z. cucurbitae* after RNAi knockdown. FAM: green fluorescence indicates the *ZcCTL-S1* probe; DAPI: blue nuclear dye. (**C**) Ovarian development status. (**D**) Total number of eggs laid. (**E**) Egg hatching rate. (**F**) Egg length. (**G**) Ovary area. Data are mean values ± SEM and were analyzed using Student’s *t*-tests. ns: not significant (*p* > 0.05). * p < 0.05, *** p < 0.001, **** p < 0.0001.

To elucidate the role of *ZcCTL-S1* in regulating reproductive processes, female *Z. cucurbitae* individuals were subjected to RNAi to specifically silence *ZcCTL-S1* expression. Compared with the ds*EGFP*-treated control group, injection of ds*CTL-S1* resulted in a significant reduction in *ZcCTL-S1* mRNA levels at 24 h (*t* = 12.9766, *df* = 3, *p* = 0.0009), 48 h (*t* = 4.5778, *df* = 3, *p* = 0.0196), and 72 h (*t* = 9.4443, *df* = 4, *p* = 0.0007) post-injection (Figure 3A). Consistent with this transcriptional downregulation, the fluorescence intensity in the trophocytes and oocytes was markedly diminished after RNAi treatment (Figure 3B).

Following interference treatment, the ovaries remained nearly spherical in shape, with a relatively compact structure and smooth surface texture; no significant morphological differences were observed between ds*CTL-S1* and ds*EGFP*-treated groups (Figure 3C). No significant difference was observed in the total number of eggs laid within three days between ds*CTL-S1* and ds*EGFP*-treated groups (*t* = 0.0928, *df* = 4, *p* = 0.9306) (Figure 3D). However, the knockdown of *ZcCTL-S1* significantly reduced overall hatchability during the three-day period (*t* = 7.8676, *df* = 16, *p* = 0.0000) (Figure 3E). In addition, both egg length (*t* = 0.3288, *df* = 37, *p* = 0.7442) and ovarian area (*t* = 0.4000, *df* = 10, *p* = 0.6976) did not differ significantly after *ZcCTL-S1* interference (Figure 3F,G).

### 3.5. miRNAs Prediction and Their Expression Patterns in Females

**Figure 4:**
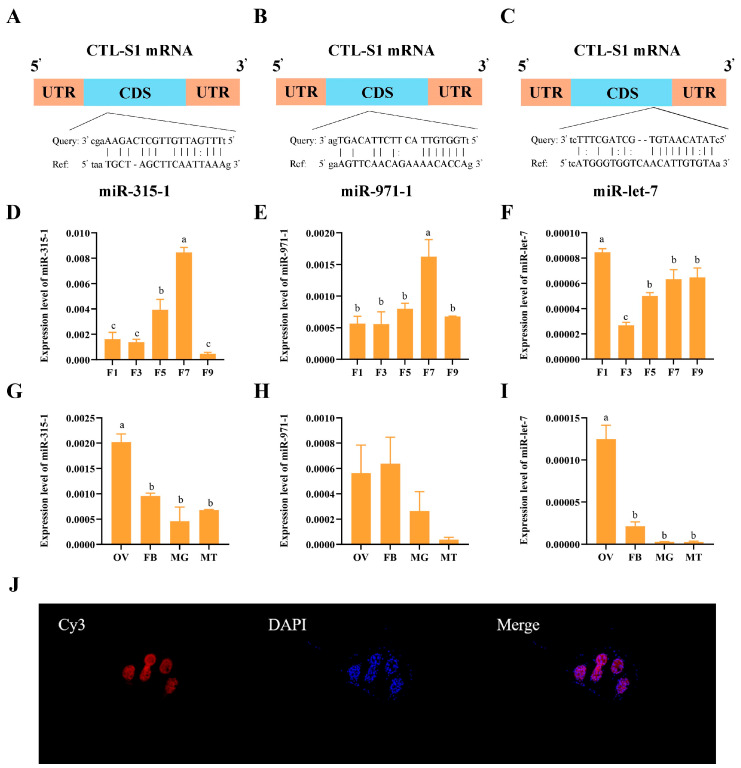
miRNA binding sites and expression patterns. (**A**–**C**) Predicted binding site of *ZcCTL-S1* with miR-315-1, miR-971-1, and miR-let-7, respectively. (**D**–**F**) Relative expression level of miR-315-1, miR-971-1, and miR-let-7 at different developmental stages, respectively. (**G**–**I**) Relative expression level of miR-315-1, miR-971-1, and miR-let-7 in different tissues, respectively. (**J**) Fluorescence in situ hybridization (FISH) localization of miR-let-7 in the ovary of *Z. cucurbitae*. Cy3: red fluorescence indicates the miR-let-7 probe; DAPI: blue nuclear dye. 1/3/5/7/9/10F: 1st/3rd/5th/7th/9th/10th instar female adult; OV: ovary; FB: fat body; MG: midgut; MT: Malpighian tubule. Data were analyzed using a one-way analysisof variance followed by Tukey’s test. Different letters indicate significant differences (*p* < 0.05).

Three candidate miRNAs (miR-let-7, miR-315-1, and miR-971-1) with predicted binding affinity to *ZcCTL-S1* were identified by combining both miRanda and RNAhybrid (Figure 4A–C). Analysis of age-dependent expression patterns in female adults revealed that miR-315-1 and miR-971-1 were significantly upregulated in 7-day-old individuals (*F*_(4,20)_ = 44.27, *p* = 0.000; *F*_(4,17)_ = 7.143, *p* = 0.001), whereas miR-let-7 exhibited peak expression levels in newly emerged (1-day-old) female adults (*F*_(4,20)_ = 17.70, *p* = 0.000) (Figure 4D–F). Meanwhile, miR-315-1 and miR-let-7 were both detected with significantly elevated expression in the ovary (*F*_(3,8)_ = 18.07, *p* = 0.001; *F*_(3,12)_ = 46.81, *p* = 0.000), whereas miR-971-1 showed no significant differences in its expression levels across various tissues (*F*_(3,8)_ = 2.653, *p* = 0.120) (Figure 4G–I). FISH assays demonstrated that miR-let-7 was detected in both trophocytes and oocytes of *Z. cucurbitae*, with its spatial distribution pattern being consistent with that of *ZcCTL-S1* (Figure 4J).

### 3.6. miRNAs Regulate the Expression of the ZcCTL-S1 Gene

**Figure 5:**
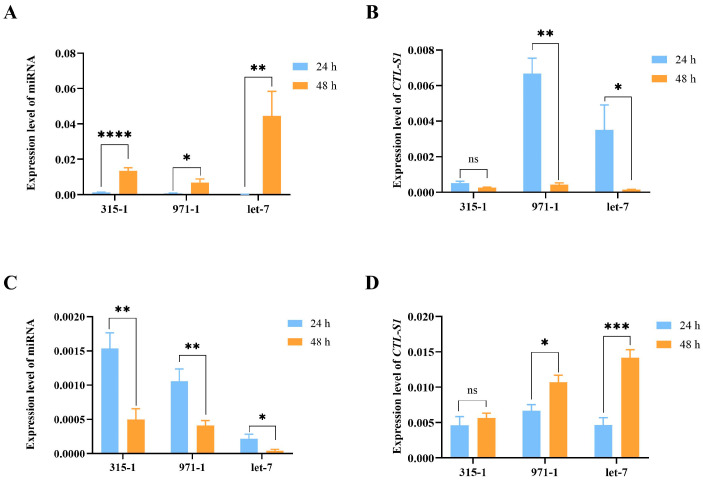
Regulatory relationship between candidate miRNAs and *ZcCTL-S1* expression in *Z. cucurbitae*. (**A**,**C**) miRNA overexpression and inhibition efficiency. (**B**,**D**) *ZcCTL-S1* expression level after miRNA overexpression and inhibition. Data are mean values ± SEM and were analyzed using Student’s *t*-tests. ns: not significant (*p* > 0.05). * p < 0.05, ** p < 0.01, *** p < 0.001, **** p < 0.0001.

Transfection with miRNA mimics led to a significant upregulation of miR-315-1, miR-971-1, and miR-let-7 expression across all treatment groups (miR-315-1: *t* = 7.356, *df* = 10, *p* < 0.001; miR-971-1: *t* = 2.877, *df* = 14, *p* = 0.012; miR-let-7: *t* = 4.055, *df* = 8, *p* = 0.004) (Figure 5A). However, a marked reduction in *ZcCTL-S1* expression was only observed in the groups treated with miR-971-1 and miR-let-7 mimics (miR-315-1: *t* = 4.948, *df* = 7, *p* = 0.002; *t* = 2.404, *df* = 10, *p* = 0.037; miR-315-1: *t* = 1.998, *df* = 7, *p* = 0.086) (Figure 5B). Treatment with miRNA inhibitors resulted in a significant downregulation of miR-315-1, miR-971-1, and miR-let-7 expression across all experimental groups (miR-315-1: *t* = 3.725, *df* = 6, *p* = 0.0098; miR-971-1: *t* = 3.683, *df* = 10, *p* = 0.0042; miR-let-7: *t* = 3.017, *df* = 8, *p* = 0.0166) (Figure 5C). Conversely, a marked upregulation of *ZcCTL-S1* expression was exclusively induced by the inhibitors targeting miR-971-1 and miR-let-7 (miR-971-1: *t* = 2.838, *df* = 7, *p* = 0.0251; miR-let-7: *t* = 6.033, *df* = 8, *p* = 0.0003; miR-315-1: *t* = 0.551, *df* = 7, *p* = 0.5987) (Figure 5D).

### 3.7. The Binding of miR-let-7 with ZcCTL-S1

**Figure 6:**
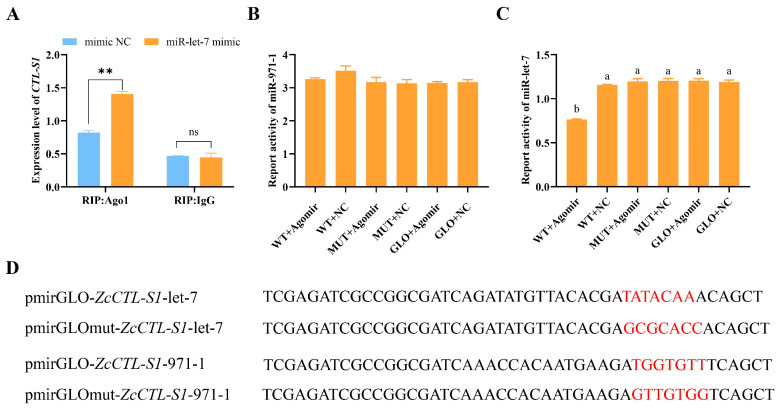
Validation of the binding between miR-let-7/miR-971-1 and *ZcCTL-S1*. (**A**) RNA immunoprecipitation assay. (**B**,**C**) Dual-luciferase reporter assay for miR-971-1 and miR-let-7. (**D**) Site-directed mutation of the pmirGLO plasmid. The red font indicates the base sequences before and after mutation. Data were analyzed using a one-way analysisof variance followed by Tukey’s test. Different letters indicate significant differences (*p* < 0.05). Data are mean values ± SEM and were analyzed using Student’s *t*-tests. ns: not significant (*p* > 0.05). ** p < 0.01.

RIP assays demonstrated that incubation of cell lysate with an AGO-1 antibody significantly increased the enrichment of *ZcCTL-S1* following miR-let-7 mimic treatment (*t* = 11.740, *df* = 3, *p* = 0.007) (Figure 6A). By contrast, no significant difference in *ZcCTL-S1* expression level was detected when the cell lysate was incubated with an IgG antibody (*t* = 0.365, *df* = 3, *p* = 0.750) (Figure 6A). These findings confirm that miR-let-7 directly targets *ZcCTL-S1* and mediates its post-transcriptional regulation.

The binding regulatory sequences of miR-971-1 and miR-let-7 were predicted to be located within the CDS regions of *ZcCTL-S1* using RNAhybrid v2.1.2 software, respectively (Figure 5B,C). To validate the roles of these binding sites in modulating *ZcCTL-S1* expression, the predicted binding motif TATACAA of *ZcCTL-S1* with miR-let-7 was mutated to GCGCACC, while the cognate binding sequence TGGTGTT of *ZcCTL-S1* with miR-971-1 was substituted with GTTGTGG (Figure 6D). We analyzed the binding of two pairs of plasmids (pmirGLO-*ZcCTL-S1-*miR-let-7 vs. pmirGLOmut-*ZcCTL-S1*-miR-let-7, and pmirGLO-*ZcCTL-S1-*miR-971-1 vs. pmirGLOmut-*ZcCTL-S1*-miR-971-1) to *ZcCTL-S1* in vitro using a dual-luciferase reporter assay. We found that no significant difference in luciferase activity was detected following transfection with the pmirGLO-*ZcCTL-S1-*miR-971-1 plasmid in combination with either the miR-971-1 mimic or mimic NC (*F*_(5,50)_ = 2.338, *p* = 0.053) (Figure 6B). This finding demonstrates that the TGGTGTT sequence within *ZcCTL-S1* does not function as a valid binding site for miR-971-1, thereby ruling out a targeted regulatory relationship between miR-971-1 and *ZcCTL-S1* (Figure 6D). In contrast, co-transfection of pmirGLO-*ZcCTL-S1-*miR-let-7 with the miR-let-7 mimic resulted in a marked decrease in luciferase activity relative to the mimic NC group (*F*_(5,66)_ = 67.488, *p* = 0.000) (Figure 6C), which verifies the presence of a miR-let-7 binding site within the TATACAA seed region of *ZcCTL-S1*. To further verify the targeted relationship, a mutated pmirGLO-*ZcCTL-S1-*mut-let-7 was co-transfected with the miR-let-7 mimic or mimic NC. No significant difference in luciferase activity was observed (*F*_(5,66)_ = 67.488, *p* = 0.000) (Figure 6C,D), supporting that *ZcCTL-S1* was the target gene of miR-let-7.

### 3.8. miR-let-7-ZcCTL-S1 Axis Regulates Reproduction

**Figure 7:**
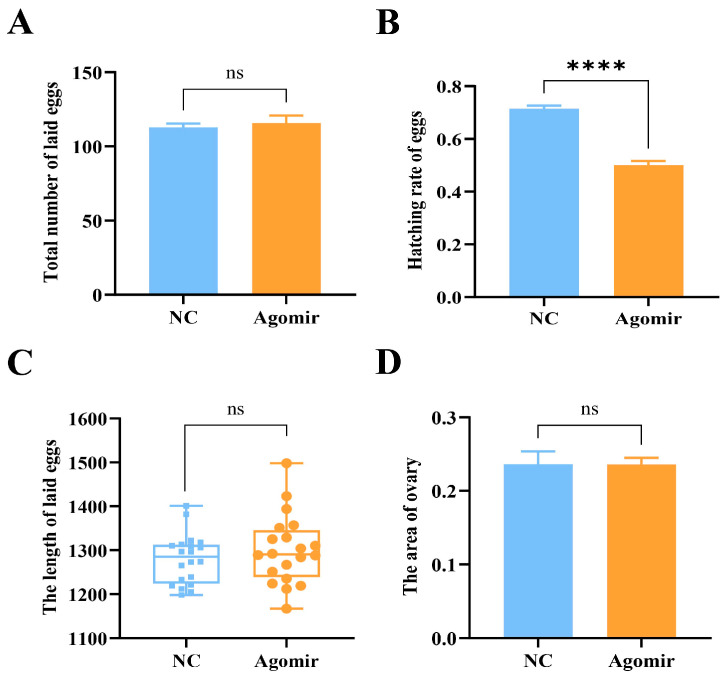
Fecundity determination of *Z. cucurbitae* after agonist injection. (**A**) Total number of eggs laid. (**B**) Egg hatching rate. (**C**) Egg length. (**D**) Ovary area. Data are mean values ± SEM and were analyzed using Student’s *t*-tests. ns: not significant (*p* > 0.05). **** p < 0.0001.

Following the identification of miR-let-7-mediated regulation of *ZcCTL-S1*, we performed overexpression to explore the role of miR-let-7 in *Z. cucurbitae* reproduction. No significant difference in total number of eggs laid (*t* = 0.496, *df* = 10, *p* = 0.631), ovary area (*t* = 0.036, *df* = 15, *p* = 0.972), and egg length (*t* = 0.986, *df* = 38, *p* = 0.330) was observed in miR-let-7 mimic group compared to mimic NC group (Figure 7A,C,D). However, a 21.33% decrease was found in hatching rate (*t* = 10.200, *df* = 10, *p* = 0.000) (Figure 7B). Collectively, the results exhibit a high consistency with the outcomes of *ZcCTL-S1* RNAi.

## 4. Discussion

RNA-Seq quantifies transcripts and their isoforms with exceptional accuracy, offering a strong approach for exploring the molecular basis of sex-specific fecundity in insects [25,26]. For instance, in *B. dorsalis* [27], *Solenopsis invicta* (Hymenoptera: Formicidae) [28], and *Plutella xylostella* (Lepidoptera: Plutellidae) [29], key differentially expressed genes (DEGs) have been identified via RNA-Seq technology, providing crucial transcriptomic evidence for elucidating the regulatory mechanisms underlying their fecundity. In this study, transcriptome analysis was performed on the ovaries, Malpighian tubules, midguts, and fat bodies of female *Z. cucurbitae*. High mapping rates were achieved for all tissues except the midgut, which aligns with previously reported mapping rates in transcriptome sequencing of male *Z. cucurbitae* and lncRNA sequencing of *Z. cucurbitae* [30,31]. Based on transcriptome analysis, we identified *ZcCTL-S1* as an ovary-specific gene in *Z. cucurbitae*.

As a galactose-specific lectin, *ZcCTL-S1* contains a conserved CLECT domain and belongs to the C-type lectin family. RT-qPCR analysis identified that *ZcCTL-S1* is strongly expressed in 5-day-old adults female and in the ovaries. This pattern was further supported by FISH assays, which indicated that the transcript is mainly localized in oocytes and trophocytes. In *A. aegypti*, *GCTL-3* is also highly expressed in ovaries and is detectable in follicle cells, and silencing *GCTL-3* reduces fecundity [32]. These observations suggest that *ZcCTL-S1* is likely involved in female reproductive processes.

C-type lectins (CTLs) are widely distributed across various insect species and are previously regarded as pivotal to pathogen recognition and immune defense [4,33]. During the immune response, CTLs can enhance the anti-infection ability of insects through multiple mechanisms, including promoting pathogen phagocytosis, regulating the synthesis of antimicrobial peptides (AMPs), and enhancing the phenoloxidase (PO) cascade reaction [34]. However, our results differ from the traditional understanding that CTLs primarily function in immune defense, as we found that RNA interference (RNAi)-mediated silencing of *ZcCTL-S1* significantly reduced the hatching rate of *Z. cucurbitae*, while egg laying was not significantly affected. This functional divergence highlights that *ZcCTL-S1* is mainly involved in regulating female reproduction rather than immune defense in *Z. cucurbitae*. Consistent with our findings, a similar pattern has been reported in *Nilaparvata lugens* (Hemiptera: Delphacidae), where knockdown of *Nllet1* (a CTL family member) led to a marked decrease in hatching rate but no significant difference in fecundity [35]. Although most previous studies have focused on the immune function of CTLs [4,33,34], an increasing number of studies have confirmed that CTLs also play crucial roles in insect reproduction. For example, silencing of *TcCTL17* in *T. castaneum* not only affected immune defense but also resulted in abnormal development and reduced fecundity [36], and in *B. mori*, specific CTLs bind to glycoproteins within the oocyte envelope to facilitate sperm–egg recognition and maintain sperm motility [7]. These studies, together with our findings, suggest that the functional diversity of CTLs is more extensive than previously recognized and that *ZcCTL-S1* is a key reproductive regulatory factor in *Z. cucurbitae*, which enriches our understanding of the non-immune functions of CTLs in insects.

To investigate the upstream miRNA of *ZcCTL-S1* in *Z. cucurbitae*, we predicted the potential target miRNAs of *ZcCTL-S1*. Among these, miR-let-7 was confirmed to exhibit a direct targeting relationship with *ZcCTL-S1*, via a combinatorial approach involving in vivo RNA immunoprecipitation, in vitro dual-luciferase reporter assays, and site-directed mutagenesis techniques. miR-let-7 exhibited high expression levels in the ovaries of adult *Z. cucurbitae*, and FISH assays further confirmed that it is predominantly localized in follicle cells. Overexpression of miR-let-7 in *Z. cucurbitae* significantly reduces the hatching rate, thereby impairing the reproductive capacity of this pest. Consistent with this, in *Locusta migratoria* (Orthoptera: Locustinae), overexpression of miR-let-7 leads to impaired ovarian development and blocked oocyte maturation [37]. In *Galeruca daurica* (Coleoptera: Chrysomelidae), let-7-5p induces ovarian development delay and reproductive diapause [38]. However, in *Drosophila* (Diptera: Drosophilidae), let-7-C is highly expressed in the head, body, and testes, but weakly expressed in the ovaries [39]. In *B. dorsalis*, let-7 shows high expression levels in the larval prothoracic gland, midgut, Malpighian tubules, and integument. Functional analysis has demonstrated that silencing let-7 disrupts normal larval pupation [40]. These findings indicate that the expression pattern of miR-let-7 varies across different species, and its biological functions in vivo are also species-specific. MicroRNAs (miRNAs) can regulate reproductive development by targeting specific genes, playing crucial roles in insects [41,42]. In the present study, miR-let-7 was demonstrated to directly target *ZcCTL-S1*, and this interaction significantly reduced the hatching rate of *Z. cucurbitae,* thereby negatively affecting reproduction. These results indicate that miRNAs participate in reproductive regulation by regulating their target genes, playing crucial roles in insects.

## 5. Conclusions

This study identified a novel regulatory module of miR-let-7 targeting the ovary-specific gene *ZcCTL-S1* in *Z.cucurbitae,* which is indispensable for female fecundity in Z. cucurbitae. This study advances our understanding of the transcriptional regulatory mechanisms governing female reproductive functions in this pest, and provides novel insights for the development of gene- or miRNA-based pest control strategies. Future studies will focus on further exploring the crosstalk between the reproductive regulation and immune functions mediated by the miR-let-7/*ZcCTL-S1* module in *Z. cucurbitae*.

## Data Availability

The data presented in this study are available on request from the corresponding author. Transcriptomic data are available in the Sequence Read Archive (SRA) of the National Center for Biotechnology Information (NCBI) under BioProject accession number PRJNA1415690 and SRA accession numbers SRR37019228–SRR37019239.

## Supplementary Materials

The following supporting information can be downloaded at https://www.mdpi.com/article/10.3390/insects17030286/s1: Table S1: Primers used for RT-qPCR analyses; Table S2: Raw data of transcriptomic sequencing results; OV, FB, MT, and MG represent the ovary, fat body, Malpighian tubules, and midgut, respectively; Figure S1. RT-qPCR validation of DEGs. OV, FB, MT, and MG represent the ovary, fat body, Malpighian tubules, and midgut, respectively; Figure S2. Amino acid sequence of the *ZcCTL-S1* gene; Figure S3. Bioinformatics analysis of the *ZcCTL-S1* gene.
