# Supplementary material for: miR-let-7 Targeting ZcCTL-S1 to Regulate Reproductive Development in Zeugodacus cucurbitae
Source: Insects. 2026 Mar 5;17(3):286. doi: 10.3390/insects17030286 (PMC13026481; doi:10.3390/insects17030286)
Supplement: Supplementary file 1 [file insects-17-00286-s001.zip › insects-4151141-supplementary.pdf]

insects

MDPI

Supplementary Materials

miR-let-7 targeting *ZcCTL-S1* to regulate reproductive development in *Zeugodacus cucurbitae*

YiKun Zhang [1,4,5,6†], GuoFeng Zhang [1,4,5,6†], Li Xiang Chen [1,4,5,6], YuXue Zhang [1,4,5,6], ShiYuan Wang [1,4,5,6], KeQing Deng [1,4,5,6], Laiwai Tun [1,4,5,6], ZhongShi Zhou [2,3*] and Lu Peng [1,4,5,6*]

Table S1. Primers used for RT-qPCR analyses.

Gene	Forward（5′ -3′）	Reverse（5′ -3′）	Purpose
LOC105208741	GAATACGGTCGCGCTTTTCC	GCCGGTGTCTTCCTCAATCA	Transcriptome validation
LOC105215561	CAAGAAGGTTGCACCAGCAC	AAATCACGTTTGGGCTGCAC	
LOC105216210	CGCAAGGCAACCGAAATCAT	GAAGGCGCCAGTACCTTCTT	
LOC105216937	TGTCGATTGGAGTGAACGCA	CAAACGATGATCGGCTGCTG	
LOC105217287	CACGTGCTATTGCTGCCATC	CAGCAATTGGTGTGGTGTCG	
LOC105217893	ACAGCTGGACCACGTTTGAT	CCGTAGATACCACCGACAGC	
LOC105218733	GTATTCCCCGTGTTCGTGGT	AGCGACGGAAAGTCTTGGTT	
LOC105219242	CGTTGGTGAGCTTGGTTGTG	CCAGGCTAATCCTACGGCTG	
LOC105219758	CGGTGGTATCGACAAGCGTA	AACCCAGGCGTACTTGAAGG	
LOC105221285	TTGTTCTACGGACTGGCGTC	AATCTGGTAGGCGGGTTTGG	
ZcCTL-S1	CTAACCAGTTACGATGCGCC	TTAACGTCGAACTGTATATCCATAACC	Gene cloning
RPL13	GTTGTGCGTTGCGAGGAATT	GCTTGTCGTATGGTGGTGGA	RT-qPCR
ZcCTL-S1	CGATGCGCCTTTTTCTCCAC	ACGTCGAACTGTATATCCATAACCT	
U6	AGGATGACACGCAAAATCGT		
miR-let-7	CTATACAATGTGCTAGCTTTCT		
miR-315-1	CTTTCGAGCAATAATTGAAACC		
miR-971-1	TTGGTGTTACTTCTTACAGTGA		
let-7 WT	CTAGCGGCCGCTAGTCTATA-CAATGTGCTAGCTTTCTG	TCGACAGAAAGCTAGCACATTGTATA-GACTAGCGGCCGCTAGAGCT	Dual-luciferase reporter assay
let-7 MUT-F	CTAGCGGCCGCTAGTCTATA-CAATGTGCTCTAGGGAG	TCGACAACCCTAGAGCACATTGTATA-GACTAGCGGCCGCTAGAGCT	
971-1 WT-F	CTAGCGGCCGCTAGTTT-GGTGTTACTTCTTACAGTGAG	TCGACTCACTGTAAGAAGTAACAC-CAAACTAGCGGCCGCTAGAGCT	
971-1 MUT-F	CTAGCGGCCGCTAGTTTGGTGTTACTTCTG-CACTGTAG	TCGACTACAGTGCAGAAGTAACAC-CAAACTAGCGGCCGCTAGAGCT	
GLO *ZcCTL-S1*	CTAGCGGCCGCTAGTCTTTCGAGCAA-TAATTGAAACCG	TCGACGGTTTCAATTATTGCTCGAAA-GACTAGCGGCCGCTAGAGCT	
ds*ZcCTL-S1*	TAATACGACTCACTATATCAA-GCTGAACTGGTATGCAG	TAATACGACTCACTATATCTCG-CAGAAATCATTATCCCACT	DsRNA synthesis
ds*EGFP*	TAATACGACTCACTATAACGACGGCAAC-TACAAGACC	TAATACGACTCACTATACTCAGGTAG-TGGTTGTCGGG	

Table S2. Raw data of transcriptomic sequencing results. OV, FB, MT and MG represent the ovary, fat body, Malpighian tubules and midgut, respectively.

sample	raw_reads	clean_reads	clean_bases	Q30	GC_pct	total_map
OV1	52731066	48318504	7.25G	97.64	42.24	42090339 (87.11%)
OV2	50703048	48291576	7.24G	97.74	43.03	43468569 (90.01%)
OV3	47909436	45371776	6.81G	97.68	43.35	40133343 (88.45%)
MT1	49176156	46901032	7.04G	97.72	40.45	41640406 (88.78%)
MT2	49336928	48732574	7.31G	96.97	43.5	36206903 (74.30%)
MT3	47876046	46181728	6.93G	97.63	40.54	40237882 (87.13%)
MG1	49312872	48116160	7.22G	96.71	42.47	24145107 (50.18%)
MG2	49163474	47370952	7.11G	97.28	40.9	16023012 (33.82%)
MG3	51801992	50963884	7.64G	96.61	41.82	24722351 (48.51%)

FB1	47997568	46557148	6.98G	96.89	44.51	38248629（82.15%）
FB2	50179346	48162768	7.22G	97.66	41.21	41980506（87.16%）
FB3	49855280	46378456	6.96G	97.79	41.68	42030886（90.63%）

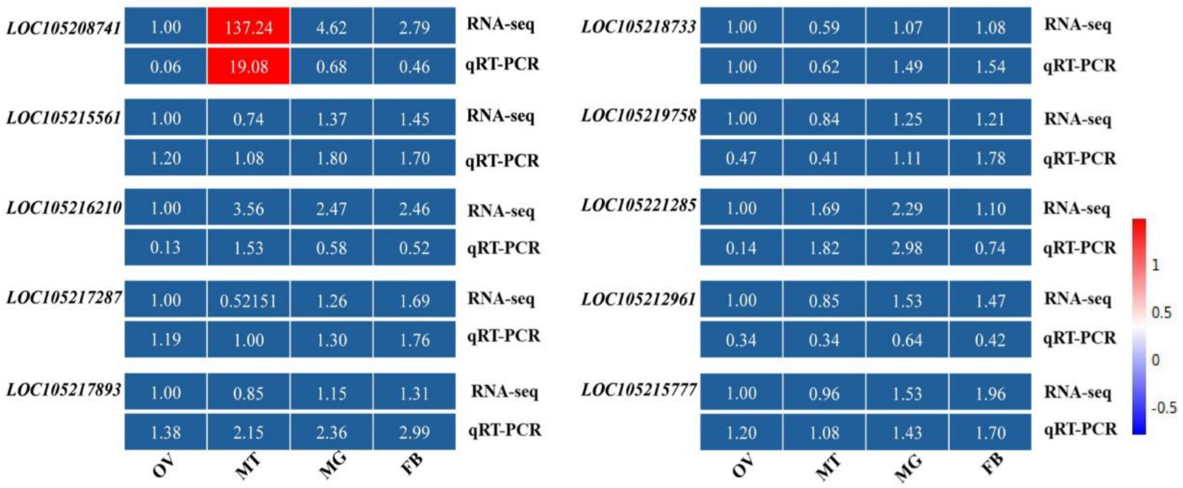

Figure S1. RT-qPCR validation of DEGs. OV, FB, MT and MG represent the ovary, fat body, Malpighian tubules and midgut, respectively.

1 ATGAAGTCTTCGAGAAAATTAATCCTGTTGCTTACAATCTTAATGCTAGCTTCAATTAAA
M K S S R K L I L L L T I L M L A S I K
61 GCAACACCTTCTACAACAGAAGTTCAACAGAAAACACCAGACGATTTTTCATACGAAGAT
A T P S T T E V Q Q K T P D D F S Y E D
121 TATTTAAATAGAACTGAGAAACGACTAACCAGTTACGATGCGCCTTTTTCTCCACTACAG
Y L N R T E K R L T S Y D A P L Q R K Y
181 CGAAAATACATCGTCACCTTAATCAAGCTGAACTGGTATGCAGCATATGCTTTCTGCGAT
I V T L I K L N W Y A A Y A F C D Q N G
241 CAAAATGGTTGGTCTCTGGCCAGCATTGAATCTACGTTGGAGCAATTTCAAGTGCAAAAC
W S L A S I E S T L E Q F Q N Y L N Y F
301 TATTTAAATTACTTTAACCTTCAAAGCAACCATTTCTGGACATCAGGTAATAAGCTCGCC
N L Q S N H F W T S G N K L A D L Q N Y
361 GACTTGCAAA ACTATCGCTGGGGTTATAATGGCTCGAAATTTAGTTATACCAATTGGGTG
R W G Y N G S K F S Y T N W V T G G P N
421 ACGGGTGGACCCAATAGTTTCATGGGTGGTCAACATTGTGTAAAATTGCAGGAGAACACT
S F M G G Q H C V K L Q E N T L K W D N
481 TTGAAGTGGGATAATGATTTCTGCGAGAATAGTCATCATTTCATATGTGAAATGAATATA
D F C E N S H H F I C E M N I D S G Y G
541 GATTCAGGTTATGGATATACAGTTCGACGTTAA
Y T V R R M N M A T

Figure S2. Amino acid sequence of the *ZcCTL-S1* gene.

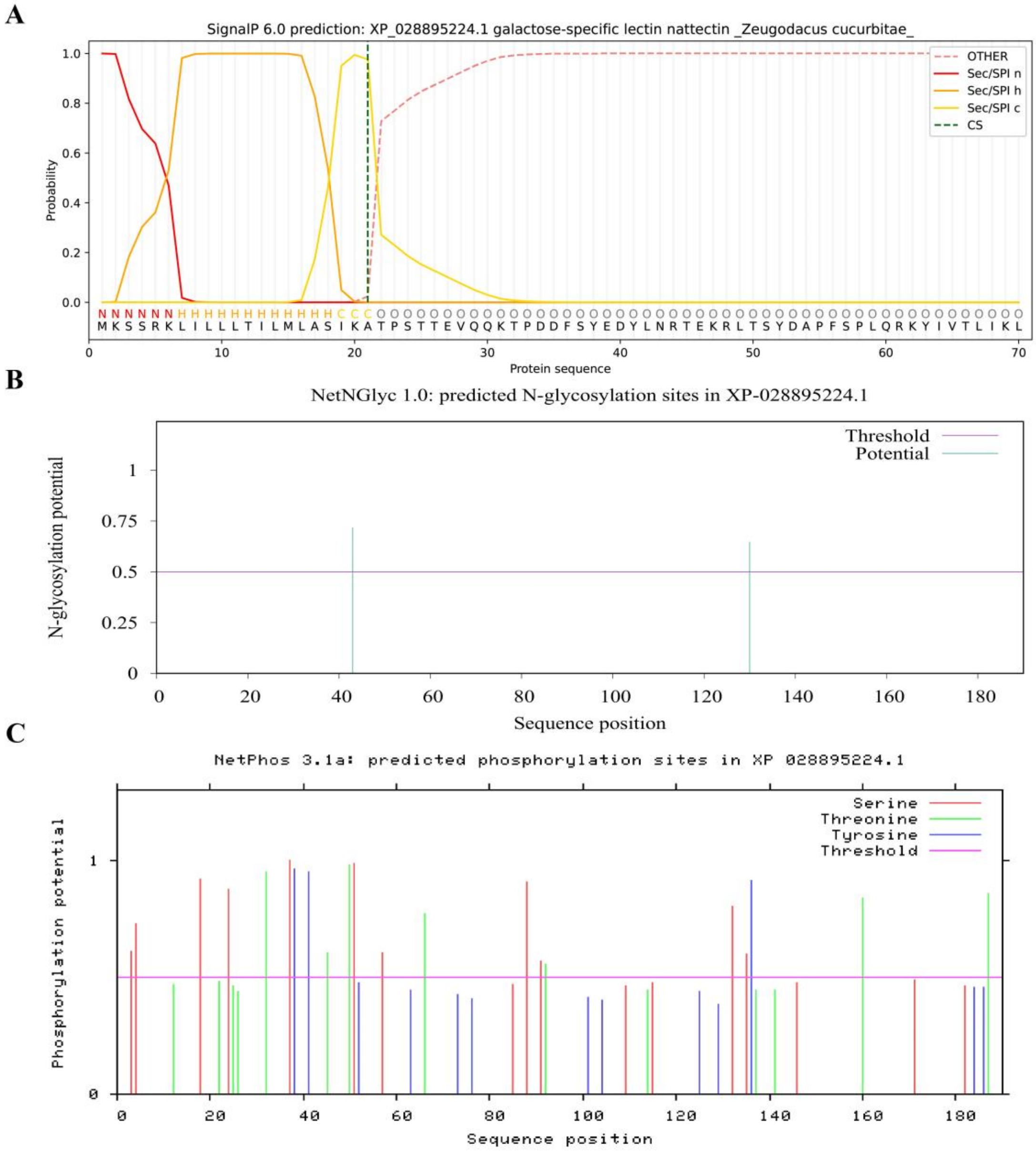

Figure S3. Bioinformatics analysis of the *ZcCTL-S1* gene. (A) The signal prediction of *ZcCTL-S1* in *Z. cucurbitae*. **(B)** The N-glycosylation sites prediction of *ZcCTL-S1* in *Z. cucurbitae.* **(C)** The phosphorylation sites prediction of *ZcCTL-S1* in *Z. cucurbitae.*
